# Redox Behavior of Anticancer Chalcone on a Glassy Carbon Electrode and Evaluation of its Interaction Parameters with DNA

**DOI:** 10.3390/ijms9081424

**Published:** 2008-08-13

**Authors:** Afzal Shah, Asad M. Khan, Rumana Qureshi, Farzana L. Ansari, Muhammad F. Nazar, Syed S. Shah

**Affiliations:** Deprtment of Chmeistry, Quaid-i-Azam University, 45320 Islamabad, Pakistan

**Keywords:** DNA, hypochromism, bathochromic shift, binding constant

## Abstract

The interaction of anticancer chalcone [AMC, 1-(4′-aminophenyl)-3-(4-*N,N*-dimethylphenyl)-2-propen-1-one] with DNA has been explored using electrochemical, spectroscopic and viscometric techniques. A shift in peak potential and decrease in peak current were observed in cyclic voltammetry and hypochromism accompanied with bathochromic shift were noticed in UV-Vis absorption spectroscopy. These findings were taken as evidence for AMC –DNA intercalation. A binding constant (*K*) with a value of 6.15 × 10^5^ M^−1^ was obtained from CV data, which was also confirmed by UV-Vis absorption titration. Moreover, the diffusion coefficient of the drug with and without DNA (D_b_ and D_u_), heterogeneous electron transfer rate constant (k^o^) and electron affinity (A) were also calculated from electrochemical data.

## 1. Introduction

Due to ineffective drugs, cancer is the secondmost leading cause of death after heart attack. Therefore, the researchers have accelerated their efforts for the generation of new anticancer drugs with high therapeutic index. In this connection, a clinically effective antitumor derivative of chalcone (calicheamicin) has stimulated the investigators to concentrate their studies on chalcones and related compounds [1].

Chalcones [2] (α,β-unsaturated ketones) are promising candidates in the new era of medicines on account of their wide spectrum of antitumor, antibacterial and anti-inflammatory activities [3–12]. Until now, several research teams have already reported the interaction of compounds and DNA using conventional three electrode cells which consisted of GCE, SCE and Pt wire. These works provide much valuable information for further investigation and the design of new anticancer drugs [13–14]. By application of spectroscopic and voltammetric techniques, parameters like binding constant, binding site size, Gibbs energy of binding, ratio of binding constants for oxidized and reduced species, diffusion coefficient (free and DNA-bound drug) and heterogeneous electron transfer rate constant can be determined.

**Scheme. 1:**
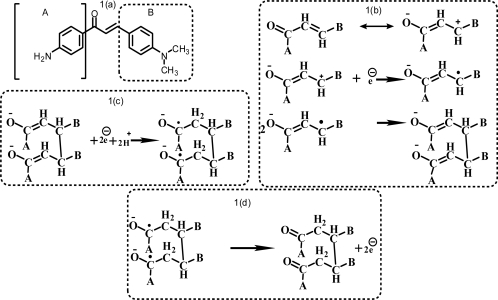
(a) Molecular structure of AMC [1-(4′-aminophenyl)-3-(4-*N,N*-dimethylphenyl)-2-propen-1-one]. (b) dimer formation AMC. (c) two electron/two proton reduction of the dimer of AMC (d) electrooxidation of the product.

The activities of several DNA binding molecules have their origin primarily in intercalation [15– 16]. However, the nature of intercalation depends upon the functional group and geometry of the molecule [17– 18]. In this paper the interaction of AMC (Scheme 1a) with DNA for unleashing its role in arresting DNA duplication has been studied by cyclic voltammetry [19–22], UV-Vis spectroscopy and viscometry at 25°C in 10% aqueous methanol buffered at pH 7.4 using 0.05 M Tris-HCl.

## 2. Results and Discussion

### 2.1. Redox studies of AMC-DNA interaction

**Figure. 1:**
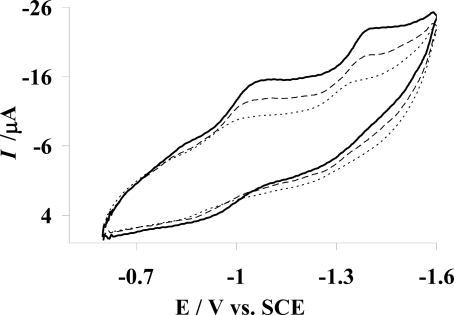
Cyclic voltammograms of 2 mM AMC in methanol with 0.1 M TBAP as supporting electrolyte in the absence (—) and presence of 1 μM DNA (– – –) and 5 μM DNA (- - -) at 100 mV/s scan rate in 0.05 M Tris-HCl buffer at 7.4 pH and 25 °C. Glassy carbon electrode (0.071 cm^2^) was used as working electrode and all potentials were reported vs. saturated calomel electrode (SCE).

The cyclic voltammetric behavior of 2 mM AMC in 0.05 M Tris-HCl buffer (pH 7.4) at GCE in the absence and presence of varying concentration of CB-DNA has been shown in Figure 1. The voltammogram (—) shows that AMC exhibits two prominent reduction peaks and one weak oxidation peak at the bare GCE. Peak I_c_ at –1.06 V corresponds to the one electron reduction of carbon 3 followed by dimer formation as shown in Scheme 1b. Peak II_c_ at –1.40 V can be attributed to the two electron/two proton reduction of the dimer as depicted in Scheme 1c.The weak anodic peak I_a_ at –0.94 V may be due to the electrooxidation of the product generated from II_c_ as illustrated in scheme 1(d).

Voltammograms (- - -) and (- - -) in Figure 1 further indicate considerable diminution of the peak currents and shift of cathodic peak potentials to less negative values by the addition of increasing concentration of DNA. Such observations can be attributed to the decrease in free drug concentration due to its intercalation into DNA [23]. The shift in peak potential indicates the interaction of AMC with DNA.

Further examination of the cyclic voltammograms reveals the disappearance of anodic peak, which may be due to the formation of electrochemically unoxidizable drug-DNA adduct. To show that the decrease in peak current is due to the formation of slowly diffusing AMC -DNA complex, the diffusion coefficient of the drug with and without DNA was determined by using Randles- Sevcik equation [24]:

(1) $$I_{p}=2.69\times 10^{5}\;n^{3/2}AC_{o}^{*}D_{o}^{1/2}v^{1/2}$$

where *I_p_* (A) is the peak current, *A* (cm^2^) is the surface area of the electrode, C_o_*(mol cm^−3^) is the bulk concentration of the electroactive species, *D_o_* (cm^2^s^−1^) is the diffusion coefficient and *ν* (Vs^−1^) is the scan rate.

**Figure. 2:**
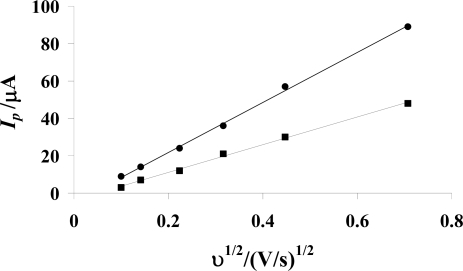
Ip vs. υ 1/2 plots of 2 mΜ AMC in the absence of DNA (•) and presence of 5 μΜ DNA (▪) at 20 mV/s (a), 50 mV/s (b), 100 mV/s (c), 200 mV/s (d) and 500 mV/s (e) in 0.05 M Tris-HCl buffer (pH 7.4) at 25 °C.

The linearity of the plot (Figure 2) between *I_p_* and *υ^1/2^* indicates that the reduction of AMC is controlled by the diffusion step. The diffusion co-efficient of 2 mM AMC in the absence and presence of 5 μM DNA was obtained as 1.11 × 10^−9^ m^2^s^−1^ and 2.97 × 10^−10^ m^2^s^−1^ from the slope of *I_p_* vs. *υ^1/2^* plot. These results indicate that the diffusion coefficient of DNA – bound drug (AMC), which is an order of magnitude lower than that of the free drug is responsible for the observed decay in peak current. Similar results have also been obtained by other investigators [25–27].

**Figure. 3:**
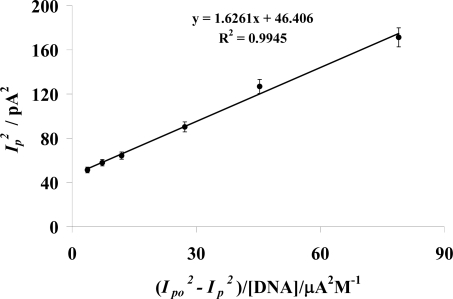
Plot of $I_{p}^{2}$ vs. ( $I_{po}^{\text{2}}-I_{p}^{2}$) / [DNA] for 2 mM AMC with varying concentration of DNA ranging from 1 μM to 50 μM in a medium buffered at pH 7.4, used to calculate the binding constant of AMC-DNA adduct. A 5% overall error in data was observed.

The binding constant (*K*) of AMC- DNA interaction was obtained by using the following equation [28–29]:

(2) $$Ip^{2}=\frac{1}{K[DNA]}(Ipo^{2}-Ip^{2})+Ipo^{2}-[\text{DNA}]$$

where *I_p_* and *I_po_* are the peak currents of AMC with and without DNA respectively. A value of 6.15 ×10^5^ M^−1^ was obtained from the slope of $I_{p}^{2}$ vs. $I_{po}^{\text{2}}-I_{p}^{2}$ / [DNA] plot as shown in Figure 3. The strong affinity of this drug to DNA as is evident from its high binding constant which could serve as an important addition to the traditional cancer treatment.

**Figure. 4:**
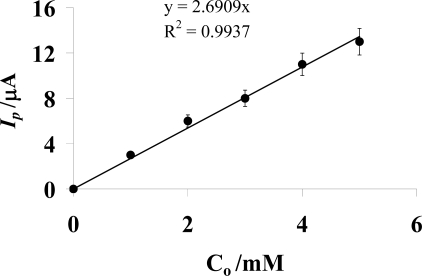
Plot of *I_p_* vs. *C_o_* used to calculate the heterogeneous electron transfer rate constant of AMC on GCE at 100 mV/s scan rate. Error bars show the fluctuation in the values of y-axis with respect to experimentally observed values.

The experimental heterogeneous electron transfer rate constant of AMC (*k^o^*) with a value of 3.95 × 10^−6^ ms^−1^ was obtained at 100 mV/s scan rate from the slope of *I_p_* vs. *C_o_* plot (Figure 4) using Reinmuth’s expression [30]:

(3) $$I_{p}=nFAC_{o}k^{o}$$

where *I_p_* and *C_o_* denote the peak current and concentration of the electroactive species respectively.

The electron affinity (A) of AMC with a value of 1.71 eV was calculated from its *E_1/2_* value by the relationship reported as [31]:

(4) $$A=\chi -E_{1/2}-S$$

where *S* is the solvation energy and χ is the work function of working electrode, whose value is 4.39 eV for glassy carbon electrode [32]. The electron affinity of AMC is comparable with 1- (4′-N,N-dimethylphenyl)-2-propen-1-one (1.69 eV) [33] but slightly greater than simple aromatic hydrocarbons [31– 34– 35] due to its conjugated carbonyl group.

The solvation energy was evaluated from Born′s equation [33]:

(5) $$S=-({\text{z}}^{2}e^{2}/2r)\;(1-1/\varepsilon \text{)}\;(1/4\pi \varepsilon_{o}\text{)}\;1/1\text{.6}x10^{-19}$$

The radius of AMC was calculated from the density equation [33]:

(6) $$\frac{4}{3}\pi r^{3}=\frac{M}{Nd}$$

### 2.2. Spectroscopic studies

**Figure. 5:**
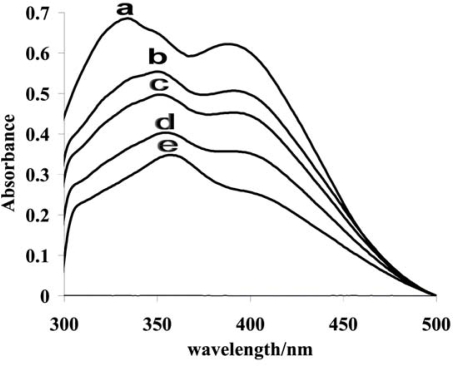
UV-Vis absorption spectra of 5 μM AMC in the absence of DNA (a), in the presence of 2.5 μM (b), 5 μM (c) 10 μ M (d) and 20 μM DNA (e) at pH 7.4 and 25 °C.

The UV–Vis absorption spectra of AMC–DNA complexes in 10% aqueous methanol maintained at pH 7.4 (0.05 M Tris-HCl buffer) were also recorded using 5 μM AMC with varying concentration (ranging from 0.5–20 μM) of DNA for getting further clues about the mode and strength of interaction. Figure 5 shows the absorption spectra of AMC in the absence and presence of DNA. The absorption spectra exhibited peculiar bathochromic and hypochromic shifts by the addition of increasing concentration of DNA. Such findings indicate the interaction of electronic states of AMC intercalating chromophore (α,β-unsaturated ketonic part) with the DNA bases, resulting in a stable AMC-DNA complex by π–π stacking and dipole–dipole interactions [36– 37]. The bathochromic shift of the drug from 336 nm to 359 nm and hypochromic effect of 0.34 after binding to a fixed amount of DNA are typical characteristics of an intercalative mode of binding [38– 39], in which the drug inserts itself between the adjacent base pairs causing partial unwinding of DNA resulting in the failure of its translation and transcription machinery. The insertion of the drug is expected to occur through minor and major grooves as its radius (0.46 nm) is less than the width (0.6 and 1.2 nm) of both these grooves [40]. The decrease in the drug absorption intensities were exploited for the evaluation of the binding constant using the following equation [41– 42]:

(7) $$\frac{A_{o}}{A-A_{o}}=\frac{\varepsilon_{u}}{\varepsilon_{b}-\varepsilon_{u}}+\frac{\varepsilon_{u}}{\varepsilon_{b}-\varepsilon_{u}}\left[\frac{1}{K[DNA]}\right]$$

where *A* and *A_o_* represent the absorbance of bound and unbound drug with ɛ_b_ and ɛ*_u_* as their respective absorption coefficients.

The binding constant with a value of 2.86 × 10^5^ M^−1^ was obtained from intercept to slope ratio of *A_0_/(A–A_0_)* vs. 1/[DNA] plot. The value of *K* obtained here is in close agreement with that obtained from CV measurements and is greater then the *K* = 2.60× .10^5^ M^−1^ obtained for the interaction of kaempferol with DNA due to a couple factors like the presence of electrophilic carcbon and extensive aromatic system [43].

### 2.3. Viscosity measurements

**Figure. 6:**
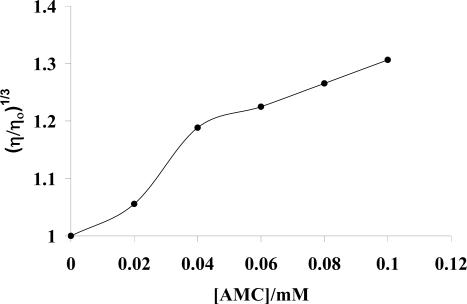
Plot of Relative viscosity (η/η_o_)^1/3^ vs. concentration of AMC in 0.05 M Tris-HCl buffer (pH 7.4) at 25 °C. Scheme 1(a) Molecular structure of AMC chemically named as 1-(4′-Aminophenyl)-3-(4-N,N-dimethylphenyl)-2-propen-1-one. (b) dimer formation AMC (c) two electron/two proton reduction of the dimer of AMC (d) electrooxidation of the product.

Electrochemical techniques generally provide necessary but insufficient evidence to support an intercalative mode of binding. Viscometric measurements can offer least ambiguous clues about the binding model in solution [13]. The change in the viscosity of 50 μM DNA upon the addition of the drug ranging from 20 μM to 100 μM is shown in Figure 6. Representing intrinsic viscosity of DNA with and without drug by η and η_o_, data were plotted as (η/η_o_)^1/3^ against the concentration of the drug. The results reveal a significant increase in the relative viscosity of DNA, indicating an intercalative mode of binding [44]. The intercalating agents for their accommodation widen the gap between the adjacent base pairs and thus cause lengthening of the double helical structure resulting in a significant increase of relative viscosity of DNA [45].

## 3. Experimental Section

### 3.1. Reagents and chemicals

AMC was kindly provided by F. L. Ansari from her combinatorial library of 120 chalcones [20– 46]. Methanol UN-no 1230 with more than 99.5% purity was used without further purification. Tetrabutylammonium perchlorate (TBAP, Fluka, 99% purity) was further purified by recrystallization using methanol as a solvent. Doubly distilled water was used to prepare all the solutions and buffered at pH 7.4 by using 0.05 M Tris-HCl buffer. DNA was extracted from chicken blood by Falcon method [21]. Stock solution of chicken blood DNA (CB-DNA) was prepared by dissolving an appropriate amount of DNA in doubly distilled water and stored at 4°C. The concentration of the stock solution of CB-DNA (200 μM in nucleotide phosphate, NP) was determined by UV absorbance at 260 nm using the molar extinction coefficient (∈) of 6600 M^−1^ cm^−1^ [22].

The nucleotide to protein (N/P) ratio of 1.85 obtained from the ratio of optical density at 260 and 280 nm (*A_260_/A_280_* = 1.85) was taken as evidence for protein free DNA [19]. Stock solution of AMC (6 mM) was prepared by dissolving AMC in 10% aqueous methanol.

### 3.2. Apparatus and procedures

Voltammetric experiments were performed using a PGSTAT 302 equipped with Autolab GPES version 4.9 (Eco Chemie, Utrecht, the Netherlands). Measurements were carried out in a conventional three electrode cell which consisted of saturated calomel electrode (SCE, Fisher Scientific Company cat no.13-639-51) as a reference electrode, a thin Pt wire of thickness 0.5 mm with an exposed end of 10 mm as the counter electrode and a bare glassy carbon electrode (GCE) with a geometric area of 0.071 cm^2^ as the working electrode. Prior to experiments, the GCE was polished with 0.25-μm diamond paste on a nylon buffing pad. For electrochemical measurements the test solution was kept in an electrochemical cell (model K64 PARC) connected to a Lauda model K-4R circulating thermostat. Absorption spectra were measured on a Shimadzu 1601 UV-Vis Spectrometer. For the extraction of DNA, a Table Top Centrifuge, Model PLC-05 (Taiwan) was used. Density and viscosity measurements were made on an Anton Paar Stabinger Viscometer SVM 3000.

For CV experiments both the concentration and volume of AMC were kept constant while varying the concentration of DNA in solution. The voltammograms were recorded as aliquots of known volume of DNA were added. The solutions were deoxygenated via purging argon gas for 10 min before every experiment and used to keep argon atmosphere throughout the measurements. All experiments were carried out at 25 °C and blood pH (7.4). Prior to every electrochemical assay the GCE was polished for carrying out the electrochemical process on a clean electrode surface.

Spectroscopic titrations were carried out for monitoring the system by keeping both the concentration and volume of the drug constant, while varying the concentration of DNA. The solutions were allowed to equilibrate for 5 min before measurements. The spectrum was recorded after each addition of DNA solution. The data were presented as the average of three experiments in all cases.

## 4. Conclusions

In general, AMC shows electrochemically, spectroscopically and vicometrically measurable interactions with DNA at blood pH and ambient temperature of 25°C. Its CV, UV-Vis and vicometric results confirmed an intercalative mode of binding with high affinity for DNA as is clear from its binding constant of 2.86 × 10^5^ M^−1^. This value is consistent with that reported for the interaction of daunomycin with DNA [47]. The Gibbs energy change (ΔG= -RTlnK) of –33.02 kJ/mol at 25°C indicate the spontaneity of the binding interaction.

The k^o^ of AMC shows an irreversible charge transfer reaction which is in accordance with the suggestions made by Matsuda and Ayabe [48]. Furthermore, the solvation energy and electron affinity fall within the range of substituted chalcones [33]. These investigations will help in further understanding of the mechanism of interaction as required for the design of new anticancer drugs.
